# Overall and Gender-Specific Effects of Intermittent Preventive Treatment of Malaria with Artemisinin-Based Combination Therapies among Schoolchildren in Mali: A Three-Group Open Label Randomized Controlled Trial

**DOI:** 10.4269/ajtmh.21-1218

**Published:** 2022-08-22

**Authors:** Hamma Maiga, Charles Opondo, R. Matthew Chico, Lauren M. Cohee, Issaka Sagara, Oumar B. Traore, Mamadou Tekete, Antoine Dara, Zoumana I. Traore, Modibo Diarra, Samba Coumare, Aly Kodio, Amadou Bamadio, Bouran Sidibe, Ogobara K. Doumbo, Abdoulaye A. Djimde

**Affiliations:** ^1^ Institut National de Santé Publique, Bamako, Mali;; ^2^ Malaria Research and Training Center, Department of Epidemiology of Parasitic Diseases, Faculty of Medicine and Dentistry, Faculty of Pharmacy, University of Sciences, Techniques and Technologies of Bamako, Bamako, Mali;; ^3^ Department of Medical Statistics, Faculty of Epidemiology and Population Health, London School of Hygiene & Tropical Medicine, London, United Kingdom;; ^4^ Department of Disease Control, Faculty of Infectious and Tropical Diseases, London School of Hygiene & Tropical Medicine, London, United Kingdom;; ^5^ Center for Vaccine Development and Global Health, University of Maryland School of Medicine, Baltimore, Maryland

## Abstract

Intermittent preventive treatment of malaria among schoolchildren (IPTsc) reduces clinical malaria, asymptomatic parasitemia, and anemia. The effects of IPTsc by gender have not been studied longitudinally. We investigated overall IPTsc efficacy and conducted a secondary analysis to explore gender-specific differences. We enrolled schoolchildren aged 6–13 years in an open-label, rolling-cohort randomized controlled trial between September 2007 and February 2013 in Kolle, Mali. Annually, schoolchildren received two full-treatment courses of sulfadoxine-pyrimethamine (SP) plus artesunate, or amodiaquine (AQ) plus artesunate, or no malaria treatment as control. We used mixed-effects generalized linear models to estimate differences in treatment outcomes across groups with interaction terms to explore gender-specific differences associated with *Plasmodium falciparum* infection, hemoglobin, and grade point averages (GPA) based on standardized testing. Overall, 305 students contributed 4,564 observations. Compared with the control, SP plus artesunate and AQ plus artesunate reduced the odds of *P. falciparum* infection (odds ratio [OR]: 0.33, 95% CI: 0.26–0.43; OR: 0.46, 95% CI: 0.36–0.59). We found strong evidence of increased mean hemoglobin concentrations (g/dL) in the SP plus artesunate group versus control (difference +0.37, 95% CI: 0.13–0.58). Collectively, schoolchildren given AQ plus artesunate had higher mean GPA (difference +0.36, 95% CI: 0.02–0.69) relative to control. Schoolgirls, compared with schoolboys, given SP plus artesunate had greater improvement in GPA (+0.50, 95% CI: −0.02 to 1.02 versus −0.27, 95% CI: −0.71 to 0.16); interaction *P* = 0.048, respectively. The IPTsc decreases *P. falciparum* infections in schoolchildren. Treatment regimens that include longer-acting drugs may be more effective at decreasing malaria-related anemia and improving educational outcomes as observed among girls in this setting.

## INTRODUCTION

In 2020, an estimated 169 million cases of malaria resulting in 444,600 deaths were reported worldwide^1^ and malaria remains one of the most serious public health problems in Africa. While malaria-attributable mortality is concentrated among children under 5 years old, the prevalence of *Plasmodium falciparum* infection relative to other age groups is highest among school-age children^2^^–^^4^ who serve as an important source of human-to-mosquito *P. falciparum* infection fuelling malaria transmission and challenging malaria elimination efforts.^5^^,^^6^

About 200 million school-age children are at risk of malaria in Africa with the prevalence of infection often exceeding 50%.^7^ Malaria infections in this age group are associated with compromised health, anemia, diminished cognitive function, and lower educational achievement.^3^^,^^7^ In Mali, *P. falciparum* infections account for 36% of medical consultations among school-aged children during the peak transmission season,^8^ and are a major cause of school absenteeism.^9^

Intermittent preventive treatment (IPT) of malaria is an intervention designed to protect at-risk populations from clinical malaria and the consequences of asymptomatic parasitemia^10^ that involves directly observed administration of full-dose antimalarial therapy at fixed intervals regardless of parasite status. For over three decades, the WHO has recommended IPT in pregnancy (IPTp) with sulfadoxine-pyrimethamine (SP) during the scheduled antenatal care visits in areas of moderate to high malaria transmission.^11^ In 2010, the WHO recommended IPT for infants (IPTi) with SP alongside routine childhood vaccination visits,^12^ and, more recently, preschool children (< 5 years) for seasonal malaria chemoprevention (SMC) with SP plus amodiaquine (AQ) in some malaria-endemic areas.^13^

There are currently no WHO recommendations, such as IPT for schoolchildren (IPTsc), despite mounting evidence that preventive therapy targeting this group decreases *P. falciparum* infections, malaria-related anemia, and improves cognitive performance among older school-age children.^14^ In both Kenya and Malawi, *P. falciparum* prevalence is higher among boys than girls, whereas the overall incidence of clinical malaria is greater among girls.^15^^,^^16^

We previously reported results from the first 8 months of an IPTsc trial.^17^ Those data showed IPTsc reduced the rates of clinical malaria, all-cause acute clinic visits, asymptomatic parasitemia, and anemia among schoolchildren.^17^^,^^18^ Here we analyze the full-trial results including 65 months of follow-up (six malaria-transmission seasons) and 4,564 observations. We report the overall effects of IPTsc and conduct a secondary analysis to explore gender-specific effects of intervention with different artemisinin-based combinations.

## MATERIALS AND METHODS

### Study setting and participants.

The study was conducted in Kolle, a subsistence farming community of approximately 3,000 inhabitants situated 57 km southwest of Bamako, Mali. The rainy season, June to October, has an average of 176 mm rainfall,^19^ when the prevalence of *P. falciparum* infection ranges from 70% to 85%,^20^ and *Anopheles gambiae sensu lato* comprises > 96% of all *Anophelines*.^19^ In contrast, there is 3 mm of rainfall during the dry season^21^ between November and May when *P. falciparum* infection prevalence is 40–50%,^20^ and 45% of all *Anophelines* are *An. funestus*.^19^ To be included in the trial, children needed to be students in the one village primary school (grades 1–6), between 6 and 13 years of age, free of severe acute illness, able to attend follow-up visits, provide written and expressed informed consent/assent (parent-only consent for 6- to 9-year-olds; parent and student for 10- to 13-year-olds), and have no history of allergy to study medications. The study clinic was staffed 24 hours a day, 7 days a week throughout the study follow-up period, and was the only medical facility in the village.

### Trial design and randomization.

The open-label, three-group, randomized controlled trial was conducted between September 2007 and February 2013 among schoolchildren who were randomized to receive IPTsc of SP plus artesunate, AQ plus artesunate, or no antimalarial therapy (control). The IPTsc was administered as two courses, 8 weeks apart from the first course early in the school term, overlapping with the high malaria transmission season (September or October). Monthly follow-up visits, including physical examinations and blood collection for measurement of hemoglobin and microscopic quantification of parasitemia, were conducted throughout the transmission season. Study outcomes were analyzed at the end of each year. Schoolchildren aged out of the trial follow-up when they completed sixth grade. Participants were randomized using a computer-generated shuffle (Microsoft Excel^©^, Microsoft Inc., Washington, WA) of study numbers and treatment group allocation. Children presenting for enrolment were assigned a study number in the sequence of presentation within their school grade. Participants were stratified by grade to ensure approximately equal numbers in each study group. Clinicians were aware of group allocation at the time of randomization. Once allocated, children remained in the same study group for the duration of the trial.

### Intervention and trial procedures.

Children received an initial history and physical examination while inclusion and exclusion criteria were reviewed. Trial staff collected finger-prick blood for the preparation of thick and thin blood smears and the measurement of hemoglobin concentration (HemoCue^©^, HemoCue Inc., Brea, CA). Monthly blood smears were read by two blinded, certified microscopists in the Malaria Research and Training Center. Discordant results were read by a third certified reader, which was considered the final read. Regardless of parasite status, students were given either: (1) SP as 25/1.25 mg/kg/day in a single-dose plus artesunate as 4 mg/kg once for 3 days; or (2) AQ as 10 mg/kg/day plus artesunate as 4 mg/kg/day for 3 days; or (3) no antimalarial treatment (control group). Vitamin C, rather than placebo, was given to first-year participants in the control group, though this was replaced by water in the following years. Children received their first course of annual study medication in September 2007, and in October in all the subsequent years. All doses were directly monitored. Participants were observed for signs of intolerance for a minimum of 30 minutes. If vomiting occurred during this observation period, treatment was readministered. Children returned the following 2 days to complete their respective regimens. Students were followed from 1 year to the next and contributed to the endpoints of the trial so long as they remained age eligible. Participants were instructed to come to the clinic for any illness between follow-up visits. Children with signs or symptoms of malaria had thick and thin smears read by microscopy and hemoglobin testing. Cases were managed with a re-dose of antimalarial treatment that matched the trial group allocation; students in the control group received SP in the first year of the trial to control SP efficacy at this time, and artemether-lumefantrine in the following years. Clinicians provided the supportive care and treatment of all other diagnoses according to national guidelines.

### Outcomes.

The study team collected outcome measures each academic year, that is, asymptomatic parasitemia, hemoglobin concentrations, and grade point average (GPA) based on standardized testing, during the monthly visits for the 5 months after the initial treatment of that year. Asymptomatic parasitemia was defined as participants having no signs of illness but at least one parasite per 100 fields on microscopy reading. The WHO definition was used for anemia defined as hemoglobin < 11g/dL.^22^ Grade point average was the numeric sum of scores on standardized monthly educational tests conducted in all schools and graded by the year group teacher, divided by the number of monthly tests for which each student sat.

### Sample size and statistical methods.

Our previous studies in Kolle found the prevalence of asymptomatic parasitemia in the control group to be 75%. To have 95% power to detect a 33% relative reduction in asymptomatic parasitemia at a 5% two-sided level of statistical significance, allowing for a 10% loss to follow-up, 97 children were required in each group for a total sample of 291. Children left the study after sixth grade; therefore, approximately 51 students were removed each year from the study by planned attrition. Data were double entered into a Microsoft Access^™^ database 2010 (Microsoft Inc., Washington, WA). Statistical analysis was performed using Stata SE version 16 (StataCorp LLC, College Station, TX). Baseline characteristics of participants were summarized by the allocation group using descriptive statistics. Pooled post-intervention outcomes aggregated by treatment group were also reported. Mixed-effects generalized linear models of the Gaussian family with an identity link were fitted to estimate differences in hemoglobin and GPA as calculated by standardized testing across allocation groups. A mixed-effects generalized linear model of the binomial family with a logit link was fitted to estimate the odds ratios for malaria prevalence across groups. In all mixed models, random intercepts were specified at the individual level with an unstructured variance–covariance matrix to allow for different correlations between pairwise measurements on the same child at different times. A negative binomial generalized linear model with robust standard errors (SE) to adjust for clustering of repeated observations at the individual level was used to estimate differences in *P. falciparum* parasite density across the allocation groups. Interaction between allocation groups and gender were further fitted in each model to investigate differences in treatment effects by gender, with a likelihood ratio test to assess evidence for a subgroup effect. All models were further adjusted for grade, which was a stratification factor in the randomization; given the strong correlation between age and grade, this also served as an adjustment for age. Additionally, the interaction between allocation group and age was explored in all models to investigate whether any treatment effects varied as children got older and progressed through grades over time. For models of hemoglobin and parasitemia, adjustments for baseline values of the outcome were also included in the models. Marginal estimates of hemoglobin predicted by the mixed-effects model with interaction were plotted against grade to visually explore trends in hemoglobin across children of different ages in the three treatment groups.

### Ethics approval and trial registration.

The study protocol was approved by the Ethics Committee of the Faculty of Medicine and Odonto-Stomatology, Faculty of Pharmacy at the University of Sciences, Techniques and Technologies of Bamako, Bamako, Mali (N°59/CE/FMPOS/2007 and amendment N°59/CE/FMPOS/2008). This trial is registered with the Pan African Clinical Trials Registry (PACTR202109827515839).

## RESULTS

**Figure 1.**
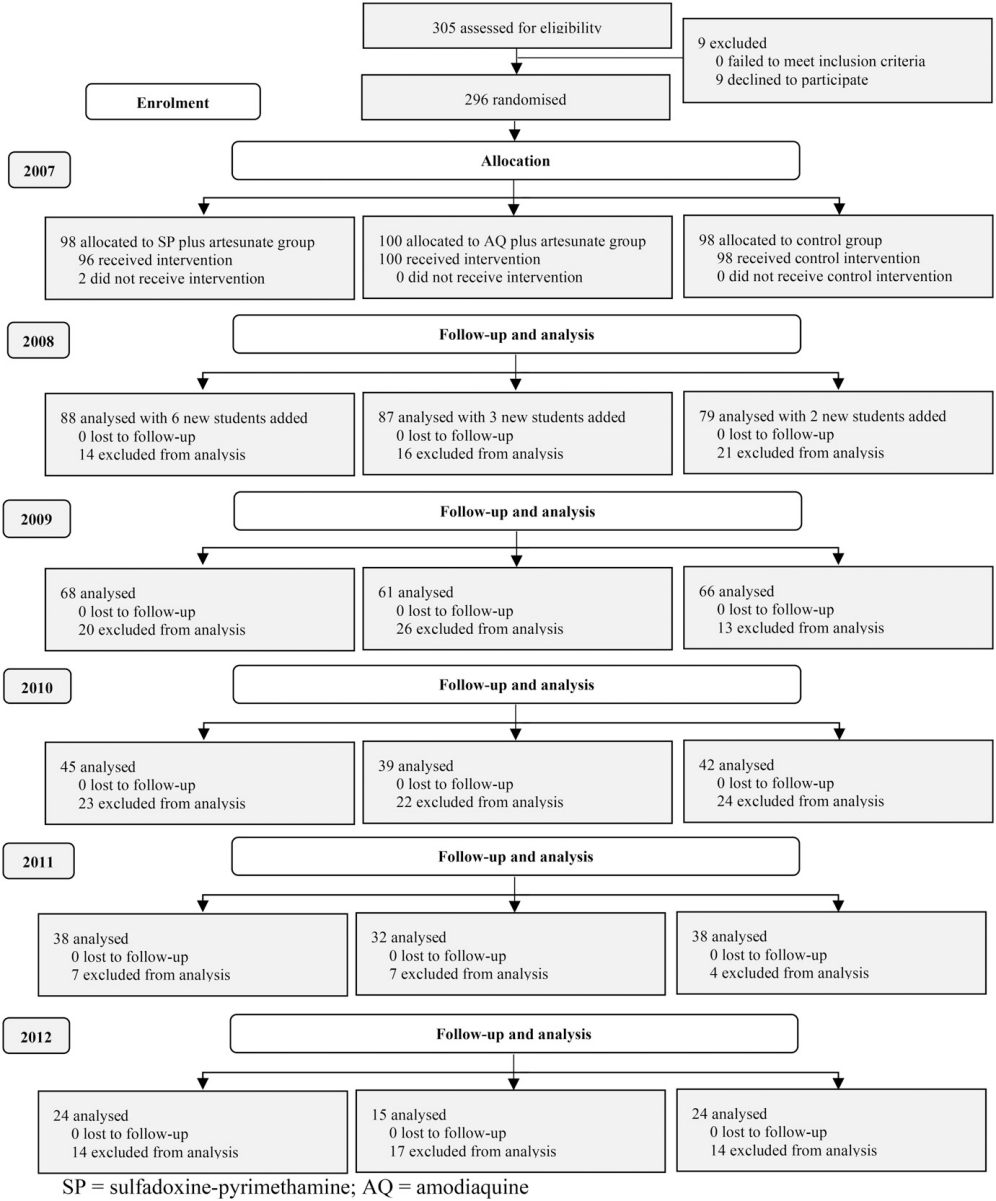
CONSORT diagram with group allocation, follow-up, and analysis of trial participants.

Among the 475 students 6–13 years of age in the village, 305 children presented for enrollment following the village crier’s announcement of the study. Nine children declined to participate and 296 enrolled in 2007. Two students assigned to SP plus artesunate (0.7%) were lost to follow-up by January 2008: one was excluded for being younger than 6 years old, and the other ceased participation at the second treatment dose. We enrolled 11 new students to add to the study cohort in October 2008. Consequently, this analysis includes 305 students: 102 received SP plus artesunate, 103 were given AQ plus artesunate, and 100 were in the control group (Figure 1).

### Participant characteristics.

Data on at least one characteristic were available from 305 children at baseline (Table 1). There were more schoolboys than schoolgirls in each allocation group and overall, perhaps reflecting a gender bias in favor of boys attending school. Children were evenly distributed across class and allocation groups. Baseline hemoglobin was similar across the groups, but the baseline prevalence of *P. falciparum* infection was higher in the AQ plus artesunate group compared with the SP plus artesunate and control groups.

**Table 1:** Baseline characteristics by study group

Characteristics	SP plus artesunate (*N* = 102)	AQ plus artesunate (*N* = 103)	Control (*N* = 100)	Overall (*N* = 305)
Gender, *n* (%)				
Male	63 (61.8)	56 (54.4)	56 (56.0)	175 (57.4)
Female	39 (38.2)	47 (45.6)	44 (44.0)	130 (42.6)
Class, *n* (%)				
1	17 (16.7)	17 (16.5)	15 (15.0)	49 (16.1)
2	17 (16.7)	18 (17.5)	18 (18.0)	53 (17.4)
3	18 (17.7)	18 (17.5)	17 (17.0)	53 (17.4)
4	17 (16.7)	17 (16.5)	15 (15.0)	49 (16.1)
5	20 (19.6)	18 (17.5)	19 (19.0)	57 (18.7)
6	13 (12.8)	15 (14.6)	16 (16.0)	44 (14.4)
*Plasmodium falciparum* prevalence, *n* (%)	4 (3.9)	8 (7.8)	5 (5.0)	17 (5.6)
*Plasmodium falciparum* parasites/μL, median (range)	0 (0–10,575)	0 (0–18,125)	0 (0–900)	0 (0–18,125)
Hemoglobin in g/dL, mean (SD)	11.4 (1.4)	11.3 (1.5)	11.2 (1.3)	11.3 (1.4)

AQ = amodiaquine; g/dL = gramme/deciliter; SP = sulfadoxine-pyrimethamine.

### Outcomes.

Prevalence of *P. falciparum* infection was measured at the end of each malaria transmission season and pooled over the 6 years of the trial by allocation group: 24.1% of children in the control group had at least one infection, compared with 10.5% given SP plus artesunate, and 13.4% who received AQ plus artesunate (adjusted OR [aOR]) of infection: 0.34 (95% CI: 0.26–0.45, *P* < 0.001) for SP plus artesunate and 0.46 (95% CI 0.36–0.59, *P* < 0.001) for AQ plus artesunate; Table 2). There was no evidence of a gender-specific or age/grade-varying difference in treatment effect on *P. falciparum* prevalence (likelihood ratio *P* value for interaction 0.169 and 0.572, respectively). There was also no evidence of an adjusted difference in *P. falciparum* parasite densities in the SP plus artesunate or AQ plus artesunate groups compared with the control group (mean parasite ratios 0.80, 95% CI: 0.49–1.31, *P* = 0.320 and 0.98, 95% CI: 0.57–1.67, *P* = 0.977) nor a gender-specific or age/grade-varying difference in effect (*P* value for interaction 0.820 and 0.101, respectively).

**Table 2:** *Plasmodium falciparum* prevalence over six consecutive malaria transmission seasons, hemoglobin levels, and GPA measured by standardized testing by study group and gender-specific effects

Outcome	Control, n* (%)	SP plus artesunate, n* (%)	AQ plus artesunate, n* (%)	Unadjusted odds ratio (95% CI) *P* value		Adjusted odds ratio (95% CI) *P* value	
				SP plus artesunate	AQ plus artesunate	SP plus artesunate	AQ plus artesunate
*P. falciparum* prevalence	1,236 (24.1%)	1,236 (10.5%)	1,198 (13.4%)	0.36 (0.28–0.47)	0.47 (0.36–0.61)	0.34 (0.26–0.45)	0.46 (0.36–0.59)
	–	–	–	*P* < 0.001	*P* < 0.001	*P* < 0.001	*P* < 0.001
Gender							
Boys	–	–	–	–	–	0.31 (0.22–0.43)	0.38 (0.27–0.52)
Girls	–	–	–	–	–	0.41 (0.26–0.63)	0.61 (0.41–0.91)
	–	–	–	–	–	*P* value for interaction = 0.169	

	Control median (range)	SP plus artesunate median (range)	AQ plus artesunate median (range)	Unadjusted rate ratio (95% CI) *P* value		Adjusted rate ratio (95% CI) *P* value	
				SP plus artesunate	AQ plus artesunate	SP plus artesunate	AQ plus artesunate
*P. falciparum* parasites/μL, density	0 (0, 72,000)	0 (0, 97,500)	0 (0, 153,500)	0.75 (0.44–1.27)	1.23 (0.69–2.19)	0.80 (0.49–1.31)	0.98 (0.57–1.67)
	–	–	–	*P* = 0.247	*P* = 0.468	*P* = 0.320	*P* = 0.977
Gender	–	–	–	–	–		
Boys	–	–	–	–	–	0.80 (0.42–1.51)	1.12 (0.57–2.17)
Girls	–	–	–	–	–	0.81 (0.37–1.76)	0.81 (0.34–1.96)
	–	–	–	–	–	*P* value for interaction = 0.820	

	Control mean (SE)	SP plus artesunate mean (SE)	AQ plus artesunate mean (SE)	Unadjusted difference (95% CI) *P* value		Adjusted difference (95% CI) *P* value	
				SP plus artesunate	AQ plus artesunate	SP plus artesunate	AQ plus artesunate
Hemoglobin in g/dL	11.9 (0.1)	12.4 (0.1)	12.1 (0.1)	0.34 (0.10–0.56)	0.11 (−0.11–0.35)	0.35 (0.13–0.58)	0.15 (−0.08–0.37)
	–	–	–	*P* = 0.004	*P* = 0.313	*P* = 0.002	*P* = 0.197
Gender	–	–	–	–	–	–	–
Boys	–	–	–	–	–	0.41 (0.11–0.70)	0.10 (−0.20–0.41)
Girls	–	–	–	–	–	0.27 (−0.08–0.62)	0.20 (−0.14–0.54)
	–	–	–	–	–	*P* value for interaction = 0.601	

	Control mean (SE)	SP plus artesunate mean (SE)	AQ plus artesunate mean (SE)	Unadjusted difference (95% CI) *P* value		Adjusted difference (95% CI) *P* value	
				SP plus artesunate	AQ plus artesunate	SP plus artesunate	AQ plus artesunate
GPA by standardized test scores	4.9 (0.1)	5.0 (0.1)	5.2 (0.1)	0.01 (−0.34 to 0.37)	0.33 (−0.02 to 0.69)	0.04 (−0.30 to 0.38)	0.36 (0.02–0.69)
	–	–	–	*P* = 0.939	*P* = 0.065	*P* = 0.816	*P* = 0.038
Gender	–	–	–	–	–	–	–
Boys	–	–	–	–	–	−0.27 (−0.71 to 0.16)	0.33 (−0.12 to 0.78)
Girls	–	–	–	–	–	0.50 (−0.02 to 1.02)	0.40 (−0.10 to 0.90)
	–	–	–	–	–	*P* value for interaction = 0.048	

AQ = amodiaquine; GPA = grade-point average; SE = standard error; SP = sulfadoxine-pyrimethamine.

• Number of repeated observations over the 6 years of follow-up.

**Figure 2.**
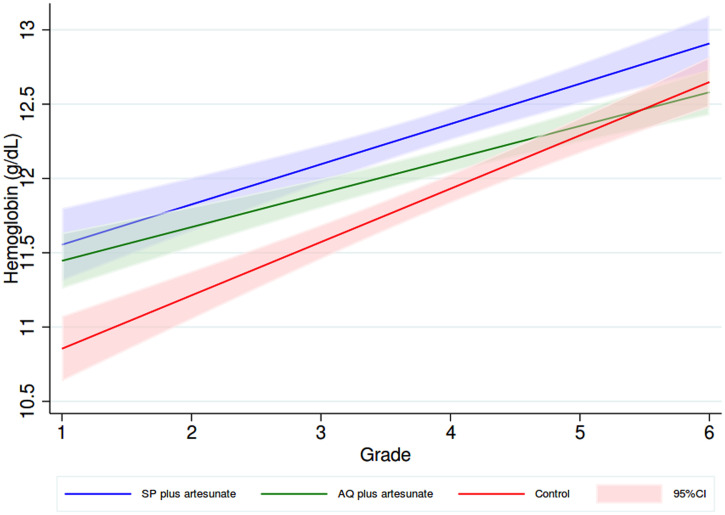
Linear trends in hemoglobin in three allocation groups by grade over the course of the study. This figure appears in color at www.ajtmh.org.

Mean hemoglobin at the end of follow-up was 11.9 g/dL (SE: 0.1) among control versus 12.4 g/dL (SE: 0.1) for those given SP plus artesunate and 12.1 g/dL (SE: 0.1) who received AQ plus artesunate, with adjusted mean differences of 0.35 g/dL (95% CI: 0.13–0.58, *P* = 0.002) in the SP plus artesunate, and 0.15 (95% CI: −0.08 to 0.37, *P* = 0.197) in the AQ plus artesunate relative to control. Nevertheless, there was evidence of a difference in treatment effect across the years of follow-up as children aged through the cohort (test of interaction between allocation group and grade in the adjusted model *P* = 0.03). Trends by grade-level over the course of the study are shown in Figure 2. The difference in hemoglobin attributed to treatment with SP plus artesunate was 0.51 g/dL (95% CI: 0.10–0.92) in children in the first grade and was attenuated every year by 0.05 g/dL (95% CI: −0.16 to 0.06) as children aged and progressed through the grades. The effect on hemoglobin attributable to treatment with AQ plus artesunate was 0.61 g/dL (95% CI: 0.20–1.03) among children in the first grade, attenuated by 0.15 g/dL (95% CI: −0.27 to −0.04) every year/grade. There was no evidence of a gender-specific difference treatment effect on mean hemoglobin (likelihood ratio *P* value for interaction 0.601).

Mean GPA at the end of follow-up was 4.9 (SE: 0.1) in the control group, 5.0 (SE: 0.1) in the SP plus artesunate group, and 5.2 (SE: 0.1) in the AQ plus artesunate group, an adjusted mean difference of 0.04 units (95% CI: −0.30 to 0.38, *P* = 0.816) among recipients of SP plus artesunate, and 0.36 (95% CI: 0.02–0.69, *P* = 0.038) among those given AQ plus artesunate, relative to control. There was some evidence of a gender-specific difference in the effect of treatment (likelihood ratio *P* value for interaction 0.048), with higher adjusted mean GPA in schoolgirls than schoolboys in the SP plus artesunate group (0.50, 95% CI: −0.02 to 1.02 versus −0.27, 95% CI: −0.71 to 0.16), but there was a similar effect by gender in the AQ plus artesunate group (0.33, 95% CI: −0.12 to 0.78 and 0.40, 95% CI: −0.10 to 0.90). There was no evidence of an age/grade-varying difference in effect on GPA (*P* value for interaction 0.93)

## DISCUSSION

Our study provides strong longitudinal evidence that preventive malaria treatment decreases *P. falciparum* infection among schoolchildren living in areas with high malaria endemicity. However, the impact of preventive treatment on anemia and education depends on the intervention drug. The antimalarial drugs used in this trial are formulations widely known to be safe and well tolerated. No cases of deaths related to the intervention or unusual adverse events were reported. These findings align with previous studies in Mali and other African countries.^14^^,^^23^^–^^25^ Our results provide evidence for the development of policy and programmatic interventions using two courses of IPTsc in areas with similar transmission patterns. In this area with high parasite prevalence^26^ and intense malaria transmission, schoolchildren need to have more total time protected against the effects of malaria infection, possibly involving chemoprophylaxis that has a long half-life such as SP.

While the study was not designed or powered to compare the drug regimens and head-to-head comparisons are not possible, differences in the efficacy of the regimens could be due to differences in drug half-life and protection from submicroscopic parasitemia. Sulfadoxine-pyrimethamine has a longer half-life than AQ.^27^ However, resistance to SP has been documented throughout much of Africa,^25^^,^^28^ suggesting its protective effect may be more compromised now, increasing the need to use alternative long-acting antimalarial drugs for preventive treatment. Because our outcome measure of infection was parasitemia detected by microscopy, we may have underestimated the impact on parasite prevalence as a high proportion of schoolchildren have submicroscopic infection.^2^ Differential drug half-lives and protection from submicroscopic infection may have limited the impact of AQ plus artesunate on anemia. Our study also supports the observation that *P. falciparum* infection, disease, and anemia indirectly affect educational achievement. Overall, AQ plus artesunate significantly increased GPA compared with control. This result aligns with a previous study that has shown a decrease in cognitive function linked to cerebral malaria and to asymptomatic parasitemia.^29^ Both *P. falciparum* disease^9^ and infection^23^ are associated with subsequent decreased cognitive function. While other clinical trials of school-based preventive treatment have also shown improvement in cognitive function, meta-analyses only showed a benefit in older school children.^14^ We did not observe this association between age and GPA but did observe attenuation of the impact of treatment on anemia over time. This may be due to decreased rates of anemia in all groups as the benefits of other school-based interventions, such as school nutrition programs and deworming campaigns accrue over years of school enrolment. However, decreasing anemia in the earliest grades may be important for their cognitive development and longer-term educational achievement.

In our study, SP plus artesunate significantly decreased *P. falciparum* malaria and anemia compared with the control without evidence of a gender-specific effect of treatment on these outcomes. However, there was evidence of a gender-specific difference in GPA by standardized testing that trended toward an increase among schoolgirls, but not schoolboys, in the SP plus artesunate group. A potential explanation is that a larger proportion of the limitation in educational achievement for girls is attributable to *P. falciparum* infection. The etiologies of anemia and decreased cognitive function are multifactorial, including malaria, helminth infections, chronic inflammation, chronic undernutrition, and micronutrient deficiencies.^30^^–^^32^ More studies are needed to evaluate further the impacts of school-based malaria chemoprevention on cognitive function and educational achievement, and to elucidate the mechanism(s) by which *P. falciparum* operates to produce these effects. Interestingly, schoolgirls with asymptomatic malaria and anemia had significantly lower GPA compared with schoolboys in the SP plus artesunate group. Gender differences in *P. falciparum* infection have been previously reported in Africa.^33^^–^^35^ Underlying reasons for such gender differences include sex-hormone-related modulation of anti-plasmodial immunity,^36^ and the lower risk of clinical malaria among boys may be attributed to the development of partial immunity as a result of repeated exposure.^37^ While this gender difference is of interest, the effects of IPTsc on *P. falciparum* prevalence and hemoglobin levels are comparable and, therefore, both genders should be considered for IPTsc in a malaria control strategy.

Our study adds to the growing body of evidence supporting IPTsc; however, there are some limitations. The study was an open label, which could have introduced bias in favor of the two treatment groups. Vitamin C was given to first-year participants in the control group, although this was replaced by water in the following years. Although vitamin C may minimally improve clinical outcomes among malaria cases,^38^ this should not have influenced our results and, if anything, would dilute our observed differences. In 2006, the WHO changed the first-line treatment of uncomplicated malaria from SP to artemisinin-based combination therapies, the protocol was amended in 2007, so that symptomatic malaria cases were then given artemether-lumefantrine. This was applied across treatment groups and, if anything, would have diluted the differences that we observed. Moreover, the half-life of artemether is 2–3 hours, whereas lumefantrine is eliminated within 3–6 days,^39^ meaning that curative treatment would confer a marginal chemoprophylactic effect. In addition, our dosing calendar was altered because of a teachers’ strike resulting in delayed dosing of several weeks in the rest of transmission seasons. While this makes the data more difficult to compare year-to-year, it does give a more practical and real-world analysis of what a school-based IPTsc program could be expected to achieve under typical circumstances. The study was performed many years ago, and changing sensitivities to study drugs might lead to different results if it was repeated now. The sample size stated in the methods was based on parasite prevalence in the control of ∼75%. However, the parasite prevalence stated in the manuscript was 1/3rd of that (24%). This could have significantly limited power to evaluate secondary outcomes, such as hemoglobin or education achievement. Also, this trial was not powered to detect differences by gender.

The high prevalence of malaria infection in schoolchildren underscores the need to reduce this burden and IPTsc is an efficacious intervention to achieve that aim. In addition, our longitudinal study supports further investigation of gender-specific effects of malaria interventions in children in sub-Saharan Africa.
